# Variability in the Diagnosis and Treatment of Interstitial Cystitis/Bladder Pain Syndrome: Internet Survey

**DOI:** 10.2196/70813

**Published:** 2025-08-05

**Authors:** Eleanora Melkonian, A Leigh Garrett, Erika Kline, Pressley Smith, Madelyn Wiesenhahn, Jeanette Petit, Alison Swierczynski, Carley Zhou, Stuart B Bauer, Rosalyn Adam, Kamil E Barbour, Sonja I Ziniel, Catherine A Brownstein

**Affiliations:** 1 APV-DTW LP, Bonita Springs, FL, United States; 2 Inspire, Arlington, VA, United States; 3 Division of Genetics and Genomics, Boston Children's Hospital, 300 Blackfan Street, Boston, MA, 02115, United States, 1 6173554764; 4 Department of Urology, Boston Children's Hospital, Boston, MA, United States; 5 Department of Surgery, Harvard Medical School, Boston, MA, United States; 6 Division of Population Health, Centers for Disease Control and Prevention, Atlanta, GA, United States; 7 Department of Pediatrics, University of Colorado School of Medicine, Aurora, CO, United States; 8 Department of Pediatrics, Harvard Medical School, Boston, MA, United States

**Keywords:** interstitial cystitis, painful bladder syndrome, social media, social networking, pain, treatment

## Abstract

**Background:**

Interstitial cystitis/bladder pain syndrome (IC/BPS) is a complex, chronic condition affecting the urinary bladder. Symptoms commonly associated with IC/BPS include painful urination, pain during intercourse, a persistent or recurrent sensation of bladder discomfort or pressure that often worsens as the bladder fills and eases after urination, urgency, frequent urination with little warning, nighttime urination disrupting sleep, and burning or other unusual urinary sensations. These symptoms can profoundly impact emotional and mental health, hinder participation in daily activities, disrupt social interactions, and strain personal relationships.

**Objective:**

This study aimed to compare the experiences of different races and ethnicities with IC/BPS regarding symptoms, diagnosis, treatment status, and treatment methods. We hypothesized that there would be differences in racial and ethnic minority groups.

**Methods:**

A cross-sectional web-based survey was administered between June and August 2022 through the Interstitial Cystitis Association and the Inspire web-based health community. Eligible adults resided in the United States, self-reported IC/BPS symptoms, and completed the survey in English. The instrument gathered demographic information, details regarding age at symptom onset, formal diagnosis status, and treatment use. Validated symptom and problem indices (the O’Leary-Sant Interstitial Cystitis Symptom Index and Problem Index) captured symptom severity and quality-of-life impact. Comparative analyses, including Fisher exact and median tests, were conducted across racial or ethnic groups (minority or multiple-race vs White and Hispanic vs non-Hispanic), and multivariable logistic regression assessed predictors of race or ethnicity on IC/BPS diagnosis status and treatment outcomes.

**Results:**

In total, 1631 individuals completed the survey. Racial or ethnic minority or multiple-race respondents constituted 11.6% (n=189) of the sample. Although IC/BPS symptom severity (Interstitial Cystitis Symptom Index or Interstitial Cystitis Problem Index scores) did not significantly differ by race or ethnicity, minority or multiple-race respondents were 50% less likely to have a formal medical diagnosis of IC/BPS than White respondents (adjusted odds ratio 0.50, 95% CI 0.30‐0.83). Overall, 86.7% (n=1408) of participants reported having received a formal IC/BPS diagnosis, and the single strongest determinant of receiving any form of treatment was having a formal diagnosis (odds ratio 29.67, 95% CI 18.32‐48.05). Over 25% (n=385) of all respondents reported using narcotic or opioid medications, indicating the challenging nature of IC/BPS symptom management.

**Conclusions:**

Minority or multiple-race participants were significantly less likely to have ever been diagnosed with IC/BPS by a health care professional, and those who were not diagnosed with IC/BPS were less likely to have used self-care behavioral and nonpharmacological treatments for their symptoms. Streamlining the diagnostic process and public health awareness campaigns outlining treatment options may help individuals manage IC/BPS symptoms.

## Introduction

Interstitial cystitis/bladder pain syndrome (IC/BPS) is a multifactorial, chronic urinary bladder syndrome. Common symptoms of IC/BPS include dysuria, dyspareunia, urgency, a persistent or recurrent sense of bladder pain or pressure that is often exacerbated as the bladder fills and is temporarily relieved after voiding, frequent urination or with little warning, nocturia that disrupts sleep, and associated burning or other abnormal urinary sensations [1]. The nature of these symptoms can significantly impair emotional well-being and psychological health [2,3], participation in daily activities [4], social functioning [5], and personal relationships [6].

Because the key symptoms of interstitial cystitis overlap with many other, more common, diagnoses, it is difficult to diagnose IC/BPS [7]. The condition may remain a diagnosis of exclusion even after careful patient history, examination, and laboratory measures [8]. IC/BPS can have a nonspecific presentation that overlaps with other urological and gynecological disorders, such as urinary tract infections, endometriosis, and vulvodynia [9]. Male population with IC/BPS are often misdiagnosed as having overactive bladder or chronic prostatitis [10-12].

Treating IC/BPS requires a multimodal approach, as no single therapy is universally effective [13]. Treatments typically involve lifestyle modifications, dietary changes, stress management, and pelvic floor physical therapy, which can help some patients but may not provide sufficient relief for others. Medications such as oral pentosan polysulfate sodium [14], antihistamines, tricyclic antidepressants, and bladder instillations with agents like lidocaine or heparin are also used, though their effectiveness varies widely and can come with side effects [15]. Some patients benefit from neuromodulation techniques like sacral nerve stimulation, while others require more invasive options such as botulinum neurotoxin injections or bladder hydrodistention [15]. While certain treatments may provide symptom relief, none consistently cure the condition. The variability in treatment effectiveness underscores the need for personalized approaches and further research into more targeted therapies.

Racial and ethnic disparities may exist within the realm of the IC/BPS research. While minority populations encompass just under 40% of the total US population [16], their representation in studies [17], including those focused on IC/BPS [18], has historically been inadequate. Emerging evidence suggests a potential variation in the natural history of IC/BPS across racial and ethnic groups. For instance, Black individuals with IC/BPS report a greater degree of urinary urgency compared to their White counterparts and are more likely to be diagnosed at a younger age [19,20].

Given the evidence of racial and ethnic differences in experiences with IC/BPS [19,20], there is a need for additional information on demographic profiles, clinical characteristics, diagnostic experiences, and patterns of treatment among minority or multiple-race individuals with IC/BPS. Therefore, we sought to comprehensively assess a large web-based population of people diagnosed with IC/BPS or “suspected to have IC/BPS based on their symptoms” for their spectrum of experiences, including demographics, symptomatology, treatments received, and quality of life.

## Methods

### Study Design and Population

We administered a cross-sectional web-based survey, focusing on multiple IC/BPS-related domains, between June and August 2022. The survey was created and administered in collaboration with the Interstitial Cystitis Association (ICA) and Inspire. ICA is a leading national nonprofit organization dedicated to advocacy, education, and support for those affected by IC/BPS. Inspire is the world’s largest web-based patient health community with over 45,000 self-identified individuals with IC/BPS.

To be eligible for study inclusion, respondents had to be members of the Inspire IC/BPS support group and discussion community or the ICA, be 18 years and older of age, be capable of responding to a web-based survey in English, be residing in the United States, and have self-reported symptoms potentially attributable to IC/BPS, regardless of having received a formal medical diagnosis of the condition. To encourage minority or multiple-race representation, recruitment materials clearly stated that minority or multiple-race individuals were invited to participate. Inspire members were invited by a message sent to their Inspire inbox. ICA members were invited by an email sent to their mailing list. Three reminder emails were sent, each 2 weeks apart.

### Ethical Considerations

The study protocol and survey content were approved by the Institutional Review Board of Boston Children’s Hospital (IRB 04-11-160). Individuals clicking through the informational page and completing the survey were considered to be consenting to the study. The participants received a US $25 e-gift card as compensation for completing the full survey. To prevent fraud, provided emails were compared to those on the mailing and membership lists, and data from unknown individuals were not included. In the case of duplicates, only the first completed survey was included in the analysis. Deidentified survey responses, with all personally identifiable information removed, are available for secondary data analysis.

### Survey Details

The survey data collected included self-reported data across key areas: (1) demographics including current age, gender identity, race and ethnicity (measured separately), highest level of education completed, current employment status, health insurance coverage status and type over the past 12 months, and presence of a direct family member with an IC/BPS diagnosis; (2) age at IC/BPS symptom onset and whether the condition had been formally diagnosed (options were “diagnosed with IC/BPS by a health care professional” or “suspected diagnosis”; the primary outcome); (3) treatment status and an inventory of the specific treatments ever used, categorized into “self-treatment” methods not requiring a prescription or clinical administration (eg, dietary modifications, over-the-counter products, and supplements) versus methods requiring a prescription or an in-office procedural treatment under medical supervision; and (4) current symptomatology using the validated O’Leary-Sant Interstitial Cystitis Symptom Index (ICSI), which grades symptom severity, as well as the corresponding Interstitial Cystitis Problem Index (ICPI), which measures the extent to which IC/BPS negatively impacts daily functional status and overall quality of life [21].

Regarding race and ethnicity, respondents were able to indicate with which of the following categories they identified: Asian, Black or African American, Hispanic or Latinx, Native American, Native Hawaiian or Pacific Islander, White, or other. Those indicating more than 1 racial category or identification with a racial category other than “White” were classified as minority or multiple-race for analysis purposes, whereas Hispanic or non-Hispanic ethnicity was recorded as a separate demographic category.

### Analysis

Descriptive statistics were used to summarize the overall demographic and clinical characteristics of the survey sample and to compare the distributions of these variables across racial or ethnic minority or multiple-race versus White respondents, as well as Hispanic versus non-Hispanic respondents, using the Fisher exact test or nonparametric median tests, as appropriate. The mean ICSI and ICPI scores were also compared between the racial or ethnic and Hispanic groups using 2-tailed *t* tests with unequal variance. Treatment rates (both overall and categorized by self-treatment vs prescription or in-office treatment) were compared across racial or ethnic and Hispanic groups using the Fisher exact test. Finally, we conducted a series of multivariable logistic regression analyses to identify factors independently associated with several key outcome variables, including having received an IC/BPS diagnosis from a health care professional and having ever received any treatment for IC/BPS symptoms, having ever used self-treatment methods, having ever required prescription or in-office treatment, and being currently treated at the time of the survey. The covariates included in these regression models were age, gender, race or ethnicity, Hispanic ethnicity, education level, employment status, health insurance, diagnosis status, family history of IC/BPS, age of symptom onset, and ICPI and ICSI scores. All statistical analyses were performed using Stata (version 16.1; StataCorp) software. The Benjamini-Hochberg adjustment was used to correct for multiple testing, with a *P* value <.02 considered statistically significant.

## Results

### Sample Characteristics

A total of 23,121 individuals were invited to the survey. Of those, the email open rate was 19.5% (4509 emails). Of the opened emails, 1631 (36%) completed the survey and formed the survey cohort. The majority of the cohort (n=1434, 87.9%) were assigned female at birth. Within the cohort, 7.2% (n=118) reported being Hispanic persons, and 11.6% (n=189) reported being members of racial minority groups: 2.3% (n=37) Asian only, 6% (n=98) Black or African American only, 0.6% (n=9) Native American or Alaskan Native only, 0.4% (n=6) Native Hawaiian or Pacific Islander only, 0.5% (n=8) other only, and 1.9% (n=31) identifying as multiple races (Table 1).

**Table 1.** Demographic characteristics of respondents by race and ethnicity^a^.

Characteristics	Overall	Race			Ethnicity		
		Minority or multiple-race	White	*P* value^b^	Hispanic	Non-Hispanic	*P* value^b^
Female (assigned gender at birth), n (%)	1434 (87.9)	132 (69.8)	1296 (90.3)	<.001	89 (75.4)	1345 (88.9)	<.001
Gender identity (n=1630), n (%)				<.001			<.001
Male	190 (11.7)	55 (29.1)	135 (9.4)		28 (23.7)	162 (10.7)	
Female	1420 (87.1)	129 (68.3)	1285 (89.6)		87 (73.7)	1333 (88.2)	
Nonbinary	11 (0.7)	1 (0.5)	10 (0.7)		1 (0.9)	10 (0.7)	
Transgender	8 (0.5)	4 (2.1)	4 (0.3)		1 (0.9)	7 (0.5)	
None of these describe me	1 (0.1)	0 (0)	1 (0.1)		1 (0.9)	0 (0)	
Age (years), median (IQR)	56 (42‐67)	42 (34‐53)	58 (44‐68)	<.001	43 (35‐55)	57 (43‐67)	<.001
Hispanic ethnicity, n (%)	118 (7.2)	47 (24.9)	65 (4.5)	<.001	118 (100)	0 (0)	<.001
Race (multiple selection possible), n (%)				<.001			
Asian only	37 (2.3)	37 (19.6)	0 (0)		0 (0)	37 (2.5)	.18
Black only	98 (6)	98 (51.9)	0 (0)		37 (33)	61 (4)	<.001
Native American or Alaskan Native only	9 (0.6)	9 (4.8)	0 (0)		3 (2.7)	6 (0.4)	.02
Native Hawaiian or Pacific Islander only	6 (0.4)	6 (3.2)	0 (0)		0 (0)	6 (0.4)	≥.99
White only	1436 (88.4)	0 (0)	1436 (100)		65 (58)	1371 (90.6)	<.001
Other only	8 (0.5)	8 (4.2)	0 (0)		5 (4.5)	3 (0.2)	<.001
Multiple-race categories	31 (1.9)	31 (16.4)	0 (0)		2 (1.8)	29 (1.9)	≥.99
Minority or multiple-race (n=1625)	189 (11.6)	189 (100)	0 (0)		47 (42)	142 (9.4)	<.001
Education (n=1628), n (%)				<.001			<.001
Some high school or less	25 (1.5)	15 (7.9)	10 (0.7)		10 (8.6)	15 (1)	
High school graduate or GED^c^	175 (10.8)	27 (14.3)	148 (10.3)		19 (16.2)	156 (10.3)	
Vocational training	81 (5)	21 (11.1)	60 (4.2)		11 (9.4)	70 (4.6)	
Associate or technical degree	240 (14.7)	26 (13.8)	210 (14.7)		19 (16.2)	221 (14.6)	
Some college	239 (14.7)	28 (14.8)	211 (14.7)		12 (10.2)	227 (15)	
Bachelor’s degree	472 (29)	44 (23.3)	428 (29.9)		27 (23.1)	445 (29.5)	
Master’s degree	296 (18.2)	19 (10.1)	276 (19.3)		15 (12.8)	281 (18.6)	
Professional degree	41 (2.5)	7 (3.7)	33 (2.3)		2 (1.7)	39 (2.6)	
Postgraduate degree	59 (3.6)	2 (1.1)	57 (4)		2 (1.7)	57 (3.8)	
Employment status (n=1578), n (%)				<.001			<.001
Full-time	508 (32.2)	75 (40.1)	431 (31.1)		51 (44)	457 (31.3)	
Part-time	218 (13.8)	33 (17.7)	183 (13.2)		20 (17.2)	198 (13.5)	
Furloughed or temporary layoff	11 (0.7)	3 (1.6)	8 (0.6)		3 (2.6)	8 (0.6)	
Maternity or medical leave	17 (1.1)	5 (2.7)	12 (0.9)		3 (2.6)	14 (1)	
Homemaker	111 (7)	14 (7.5)	97 (7)		7 (6)	104 (7.1)	
Student	21 (1.3)	3 (1.6)	18 (1.3)		1 (0.9)	20 (1.4)	
Unemployed on disability	145 (9.2)	22 (11.8)	122 (8.8)		8 (6.9)	137 (9.4)	
Unemployed	84 (5.3)	13 (7)	70 (5.1)		12 (10.3)	72 (4.9)	
Retired	463 (29.3)	19 (10.2)	444 (32.1)		11 (9.5)	452 (30.9)	
Health insurance for past 12 months (n=1498), n (%)	1431 (95.5)	148 (89.2)	1278 (96.3)	<.001	85 (87.6)	1346 (96.1)	.001
Health insurance (n=1543), n (%)				<.001			<.001
Through employer	679 (44)	71 (41.8)	605 (44.2)		49 (49.5)	630 (43.6)	
Purchase directly from insurance	165 (10.7)	41 (24.1)	124 (9.1)		18 (18.2)	147 (10.2)	
Medicare	467 (30.3)	24 (14.1)	443 (32.4)		12 (12.1)	455 (31.5)	
Medicaid	(124 (8)	27 (15.9)	97 (7.1)		14 (14.1)	110 (7.6)	
Tricare, VA, Indian Health Service, other	108 (7)	7 (4.1)	99 (7.2)		6 (6.1)	102 (7.1)	
Diagnosis of IC/BPS^d^ by health care professional, n (%)	1408 (86.7)	143 (75.7)	1265 (88.1)	<.001	93 (78.8)	1321 (87.3)	.02
Age at symptom onset (n=1594) (years), n (%)				<.001			<.001
<30	547 (34.3)	63 (33.7)	482 (34.4)		43 (37.1)	504 (34.1)	
30‐39	352 (22.1)	57 (30.5)	292 (20.8)		37 (31.9)	315 (21.3)	
40‐49	279 (17.5)	40 (21.4)	239 (17.1)		24 (20.7)	255 (17.3)	
50‐59	231 (14.5)	22 (11.8)	208 (14.9)		9 (7.8)	222 (15)	
60+	185 (11.6)	5 (2.7)	180 (12.9)		3 (2.6)	182 (12.3)	
Direct relative with IC/BPS diagnosis^e^, n (%)	160 (9.8)	17 (9)	143 (10)	.80	11 (9.3)	149 (9.9)	≥.99
ICSI^f^				.08			.27
Values, n (%)	1411 (100)	175 (12.4)	1236 (87.6)		108 (7.7)	1303 (92.3)	
Mean (SD)	11.5 (4.12)	11 (3.57)	11.5 (4.19)		11.1 (3.78)	11.5 (4.14)	
ICPI^g^				.37			.55
Values, n (%)	1551 (100)	184 (11.9)	1367 (88.1)		114 (7.4)	1437 (92.6)	
Mean (SD)	10.7 (3.58)	10.9 (3.24)	10.7 (3.62)		10.9 (3.28)	10.7 (3.60)	

a This table presents the demographic characteristics of the study population (n=1631), stratified by race and ethnicity. Key variables include gender identity, age, educational attainment, employment status, health insurance coverage, and diagnosis-related factors. Comparisons between racial and ethnic groups were conducted using Fisher exact tests for categorical variables and median tests for the continuous variable, age. Significant differences (*P* values) highlight variations across racial and ethnic groups, providing insights into disparities in demographics, socioeconomic factors, and health care access among respondents.

b Fisher exact tests were used for categorical variables, while a median test was used for the continuous variable, age.

c GED: general equivalency diploma.

d IC/BPS: interstitial cystitis/bladder pain syndrome.

e A direct relative was defined as a blood relative including parents, siblings, or children.

f ICSI: Interstitial Cystitis Symptom Index.

g ICPI: Interstitial Cystitis Problem Index.

Compared to White respondents, those classified as minority or multiple-race tended to be younger (median 42, IQR 34‐53 years vs median 58, IQR 44‐68 years; *P*<.001) and had lower levels of educational attainment (*P*<.001). Hispanic respondents reported an earlier age of initial symptom onset than their non-Hispanic counterparts (69% (n=80) vs 55.4% (n=819) before age 40 years; *P*<.001). No statistically significant differences were observed in the mean ICSI urinary symptom scores or ICPI quality-of-life impact scores across the racial or ethnic and Hispanic groups, suggesting a similar level of overall symptom burden and condition severity between the populations (Table 1).

Over 85% (n=1408, 86.7%) of respondents were diagnosed with IC/BPS by a health care professional; the remaining 13.3% (n=223) reported having a “suspected diagnosis” of IC/BPS.

Respondents having a “suspected diagnosis” of IC/BPS reported most of the classic symptoms associated with IC/BPS but to a lesser degree versus those diagnosed by a health care provider. Significantly more individuals diagnosed with IC/BPS self-reported currently having any IC/BPS symptoms (98.1%, n=1381) based on the ICPI than those with a suspected diagnosis (92.5%, n=206); *P*<.001): frequent urination during the day, getting up at night to urinate, needing to urinate with little warning, and burning, pain, discomfort, or pressure in the bladder. The mean and median ICSI were also significantly lower in those suspected of having IC/BPS than in those diagnosed: 10.31 (SD 3.52) versus 11.59 (SD 4.17) and 10 (IQR 8‐13) versus 11 (IQR 9‐15), respectively; both *P*<.001. The same was observed for the mean and median ICPI: 9.84 (SD 3.46) versus 10.85 (SD 3.58) and 10 (IQR 7‐13) versus 11 (IQR 8‐14), respectively; both *P*<.001.

### Treatment Use

When queried about treatments received for IC/BPS symptoms, over 90% of respondents reported having used some form of therapy, with a median of 6 (IQR 4-10) treatment types tried by each participant (Table 2). However, treatment rates were significantly lower among racial or ethnic minority or multiple-race respondents than among White counterparts (n=151, 79.9% vs n=1317, 92.6%; *P*<.001) and among Hispanic versus non-Hispanic respondents (n=91, 77.8% vs n=1383, 92.1%; *P*<.001). Minority or multiple-race participants reported using fewer cumulative treatment types than their White and non-Hispanic counterparts: medians of 4 (IQR 3‐7) versus 7 (IQR 4‐10; minority or multiple-race vs White: *P*<.001) and 5 (IQR 3‐8) versus 6 (IQR 4‐10) types (Hispanic vs non-Hispanic: *P*=.003).

**Table 2.** Treatment use among respondents by treatment type, race, and ethnicity^a^.

Treatment	Overall	By race (n=1612)			By ethnicity (n=1618)		
		Minority or multiple-race (n=151)	White (n=1423)	*P* value^b^	Hispanic (n=117)	Non-Hispanic (n=1501)	*P* value^b^
Any (n=1618), n (%)	1474 (91.1)	151 (79.9)	1317 (92.6)	<.001	91 (77.8)	1383 (92.1)	<.001
Number of treatment types ever used (n=1474), median (IQR)	6 (4-10)	4 (3-7)	7 (4-10)	<.001	5 (3-8)	6 (4-10)	.003
Treatment type categories (n=1474), n (%)							
Self-treatment	1313 (89.4)	127 (84.1)	1186 (90.1)	.04^c^	75 (82.4)	1243 (89.9)	.03^c^
Prescription or in-office treatment	1195 (90.9)	140 (92.7)	1195 (90.7)	.55	83 (91.2)	1257 (90.9)	≥.99
Treatment types (n=1474), n (%)^d^							
IC^e^ diet, modified or elimination diet	1079 (73.2)	79 (52.3)	995 (75.6)	<.001	54 (59.3)	1025 (74.1)	.003
Over-the-counter products	780 (52.9)	38 (25.2)	737 (56)	<.001	38 (41.8)	742 (53.7)	.03^c^
Anti-inflammatory medications	739 (50.1)	68 (45)	669 (50.8)	.20	43 (47.3)	696 (50.3)	.59
Antidepressants	731 (49.6)	44 (29.1)	683 (51.9)	<.001	37 (40.7)	694 (50.2)	.08
Bladder instillations	696 (47.2)	50 (33.1)	642 (48.8)	<.001	40 (44)	656 (47.4)	.59
Antihistamines	647 (43.9)	47 (31.1)	597 (45.3)	.001	27 (29.7)	620 (44.8)	.005
Pelvic floor PT^f^	618 (41.9)	45 (29.8)	569 (43.2)	.002	33 (36.3)	585 (42.3)	.28
Bladder hydrodistention	526 (35.7)	36 (23.8)	487 (37)	.001	23 (25.3)	503 (36.4)	.03^c^
Pentosan polysulfate sodium	521 (35.4)	35 (23.2)	483 (36.7)	.001	25 (27.5)	496 (35.9)	.11
Nonnarcotic pain medication	500 (33.9)	26 (17.2)	472 (35.8)	<.001	18 (19.8)	482 (34.9)	.003
Supplements	464 (31.5)	24 (15.9)	438 (33.3)	<.001	24 (26.4)	440 (31.8)	.30
Narcotics or opioids	385 (26.1)	33 (21.9)	350 (26.6)	.24	16 (17.6)	369 (26.7)	.06
Topical medications	365 (24.8)	38 (25.2)	327 (24.8)	.92	17 (18.7)	348 (25.2)	.21
Histamine blockers	364 (24.7)	28 (18.5)	336 (25.5)	.07	15 (16.5)	349 (25.2)	.06
Antispasmodics	318 (21.6)	28 (18.5)	289 (21.9)	.40	20 (22)	298 (21.6)	.90
Acupuncture	279 (18.9)	26 (17.2)	253 (19.2)	.66	15 (16.5)	264 (19.1)	.68
Cannabinoids	266 (18.1)	24 (15.9)	242 (18.4)	.50	8 (8.8)	258 (18.7)	.02
Biofeedback	145 (9.8)	15 (9.9)	130 (9.9)	≥.99	7 (7.7)	138 (10)	.59
Neurostimulation	140 (9.5)	14 (9.3)	126 (9.6)	≥.99	8 (8.8)	132 (9.5)	≥.99
Botulinum neurotoxin	139 (9.4)	17 (11.3)	122 (9.3)	.46	6 (6.6)	133 (9.6)	.49
Percutaneous tibial nerve stimulation	132 (9)	14 (9.3)	118 (9)	.88	8 (8.8)	124 (9)	≥.99
Laser	117 (7.9)	12 (8)	105 (8)	≥.99	9 (9.9)	108 (7.8)	.43
Surgery	102 (6.9)	10 (6.6)	92 (7)	≥.99	5 (5.5)	97 (7)	.83
Triamcinolone	98 (6.7)	13 (8.6)	85 (6.5)	.30	9 (9.9)	89 (6.4)	.19
Immunosuppressants	52 (3.5)	13 (8.6)	39 (3)	.002	3 (3.3)	49 (3.5)	≥.99
None of these	6 (0.4)	1 (0.7)	5 (0.4)	.48	1 (1.1)	5 (0.4)	.32
Other	270 (18.3)	12 (8)	254 (19.3)	<.001	18 (19.8)	252 (18.2)	.68
Currently treated (n=1618), n (%)	1015 (62.7)	112 (59.3)	897 (63)	.34	60 (51.3)	955 (63.6)	.01

a This table summarizes the treatment use patterns among study participants (n=1618), stratified by race and ethnicity. The table presents the proportion of respondents who reported ever using any treatment, the median number of treatment types used, and the distribution of specific treatment modalities, including self-treatment, prescription or in-office treatments, and various pharmacologic and nonpharmacologic interventions. Treatment types are listed in descending order of overall use. Comparisons between racial and ethnic groups were conducted using Fisher exact tests for categorical variables and median tests for continuous variables. Statistically significant *P* values indicate disparities in treatment access and use across demographic groups. The Benjamini-Hochberg adjustment was applied to account for multiple comparisons. These findings highlight potential differences in treatment access and preferences among diverse patient populations.

b Fisher exact tests were used for categorical variables, while a median test was used for the continuous variable, age.

c Treatment types are ordered from most common to least common among the overall sample population.

d This *P* value is no longer statistically significant due to the new corrected significance level (*P*<.02) after the Benjamini-Hochberg adjustment for multiple testing.

e IC: interstitial cystitis.

f PT: physical therapy.

Of all respondents, 89.4% (n=1313) indicated using behavioral and nonpharmacological treatments, such as dietary modifications and over-the-counter products, which were self-implemented. Likewise, 90.9% (n=1195) reported using treatments requiring clinical supervision such as prescription medications, instillations, pelvic floor physical therapy, interventional procedures, and other provider-administered therapies.

Significant differences in treatment rates for minority or multiple-race versus White respondents were observed for the use of dietary modifications such as elimination or restrictive IC/BPS diets (n=79, 52.3% vs n=995, 75.6%; *P*<.001), over-the-counter products (n=38, 25.2% vs n=737, 56%; *P*<.001), antidepressants (n=44, 29.1% vs n=683, 51.9%; *P*<.001), bladder instillations (n=50, 33.1% vs n=642, 48.8%; *P*<.001), antihistamines (n=47, 31.1% vs n=597, 45.3%; *P*=.001), pelvic floor physical therapy (n=45, 29.8% vs n=569, 43.2%; *P*=.002), bladder hydrodistention (n=36, 23.8% vs n=487, 37%; *P*=.001), pentosan polysulfate sodium (n=35, 23.2% vs n=483, 36.7%; *P*=.001), nonnarcotic pain medications (n=26, 17.2% vs n=472, 35.8%; *P*<.001), and nutraceuticals and other supplements (n=24, 15.9% vs n=438, 33.3%; *P*<.001). Minority or multiple-race respondents reported significantly higher rates of immunosuppressant use than White respondents (n=13, 8.6% vs n=39, 3%; *P*=.002).

Significant differences in treatment rates for Hispanic versus non-Hispanic respondents were observed for the use of dietary modifications, such as elimination or restrictive IC/BPS diets (n=54, 59.3% vs n=1025, 74.1%; *P*<=.003), antihistamines (n=27, 29.7% vs n=620, 44.8%; *P*=.005), and nonnarcotic pain medications (n=18, 19.8% vs n=482, 34.9%; *P*=.003). There was also a significant difference in those being currently treated, among Hispanic and non-Hispanic respondents (n=60, 51.3% vs n=955, 63.6%; *P*=.01).

No significant difference was identified in the use of narcotics or opioids, with 26.1% (n=385) of surveyed individuals reporting using them, and no significant difference by race (minority or multiple-race: 21.9% (n=33) vs White: 26.6% (n=350); *P*=.24) or ethnicity (Hispanic: 17.6% (n= 16) vs non-Hispanic: 26.7% (n=369); *P*=.06)

### Multivariable Analyses

After controlling for age, gender, socioeconomic status, and other relevant factors, the odds of having been diagnosed with IC/BPS by a health care professional were 50% lower among racial or ethnic minority or multiple-race respondents compared to their White counterparts (odds ratio [OR] 0.50, 95% CI 0.30‐0.83; Table 3). A similar magnitude difference in diagnostic likelihood was observed between Hispanic and non-Hispanic respondents in adjusted analyses but did not reach statistical significance (OR 0.56, 95% CI 0.31‐1.03). Other factors associated with higher odds of having received a diagnosis included assigned female at birth (OR 2.87, 95% CI 1.82‐4.55 vs male) and an earlier age of symptom onset. Those experiencing initial symptoms at an older age reported having a lower likelihood of diagnosis compared to those with onset by age 29 years (ORs ranging from 0.01 for onset at age 60+ years to 0.24 for onset at age 30‐39 years).

**Table 3.** Multivariable logistic regression models predicting interstitial cystitis/bladder pain syndrome (IC/BPS) diagnosis and treatment use^a^.

	Diagnosed with IC/BPS^b^		Ever been treated in any way^c^		Ever self-treated^c^		Ever received prescription or in-office treatment^c^		Currently treated^d^	
	OR^e^ (95% CI)	*P* value	OR (95% CI)	*P* value	OR (95% CI)	*P* value	OR (95% CI)	*P* value	OR (95% CI)	*P* value
Hispanic (comparison group: non-Hispanic)	0.56 (0.31‐1.03)	.06	0.64 (0.30‐1.33)	.23	0.54 (0.29‐1.01)	.05	0.88 (0.53‐2.12)	.75	0.75 (0.46‐1.25)	.27
Minority or multiple-race (comparison group: White)	0.50 (0.30‐0.83)	.008	0.93 (0.51‐1.71)	.82	0.61 (0.36‐1.03)	.06	1.06 (0.53‐2.12)	.86	1.12 (0.74‐1.69)	.60
Female (gender assigned at birth; comparison group: male)	2.87 (1.82‐4.55)	.001	2.24 (1.31‐3.83)	.003	1.88 (1.14‐3.10)	.01	1.06 (0.65‐2.16)	.85	1.35 (0.91‐2.00)	.14
Age (years)	1.08 (1.05‐1.11)	<.001	1.00 (0.99‐1.02)	.68	0.98 (0.97‐1.00)	.01	0.88 (0.38‐2.00)	.11	0.98 (0.97‐1.00)	.007
Education (comparison group: high school graduate or GED^f^ or less)										
Vocational training, associate or technical degree, some college	1.08 (0.57‐2.05)	.82	1.24 (0.66‐2.36)	.51	1.21 (0.71‐2.04)	.48	0.95 (0.51‐1.76)	.86	1.04 (0.69‐1.57)	.86
Bachelor’s degree	0.95 (0.49‐1.87)	.89	1.26 (0.65‐2.46)	.50	1.69 (0.96‐2.99)	.07	1.41 (0.73‐2.74)	.31	1.51 (0.98‐2.33)	.06
Master’s degree or higher	0.74 (0.37‐1.48)	.40	3.34 (1.51‐7.39)	.003	2.07 (1.13‐3.78)	.02	1.46 (0.74‐2.89)	.28	1.42 (0.91‐2.23)	.13
Current employment status (comparison group: full-time employed)										
Part-time employed	—^g^	—	—	—	—	—	—	—	0.65 (0.44‐0.97)	.03^h^
Retired	—	—	—	—	—	—	—	—	0.93 (0.62‐1.39)	.71
Unemployed	—	—	—	—	—	—	—	—	0.54 (0.31‐0.94)	.03^h^
Medical leave or disability	—	—	—	—	—	—	—	—	1.14 (0.71‐1.82)	.59
Homemaker or student	—	—	—	—	—	—	—	—	0.43 (0.27‐0.69)	<.001
Continuous health insurance during past 12 months	—	—	—	—	—	—	—	—	1.08 (0.60‐1.94)	.79
Diagnosed with IC/BPS by health care provider	—	—	29.67 (18.32‐48.05)	<.001	2.36 (1.10‐5.06)	.03^f^	3.99 (1.95‐8.16)	<.001	5.55 (3.41‐9.04)	<.001
Direct relative with IC/BPS diagnosis	1.80 (0.84‐3.89)	.13	—	—	—	—	—	—	—	—
Age at symptom onset (years) (comparison group:<30 years)										
30‐39	0.24 (0.12‐0.46)	<.001	—	—	—	—	—	—	—	—
40‐49	0.09 (0.04‐0.20)	<.001	—	—	—	—	—	—	—	—
50‐59	0.09 (0.03‐0.26)	<.001	—	—	—	—	—	—	—	—
60+	0.01 (0.004‐0.05)	<.001	—	—	—	—	—	—	—	—
ICPI^b^^,i^	—	—	—	—	—	—	—	—	1.02 (0.97‐1.08)	.48
ICSI^b^^,j^	—	—	—	—	—	—	—	—	1.01 (0.96‐1.06)	.79

a This table presents the results of multivariable logistic regression models examining the associations between demographic factors and the likelihood of being diagnosed with IC/BPS as well as different forms of treatment use. Odds ratios (ORs) with 95% CIs are reported for key predictors, including race, ethnicity, gender assigned at birth, age, education, employment status, insurance coverage, family history of IC/BPS, and age at symptom onset. The table includes five regression models: (1) diagnosis of IC/BPS by a health care provider, (2) ever having received any treatment, (3) self-treatment, (4) prescription or in-office treatment, and (5) current treatment. Significant findings indicate disparities in IC/BPS diagnosis and treatment use across racial and ethnic groups as well as differences based on socioeconomic and health care access factors. Notably, being of minority or multiple-race status was associated with lower odds of receiving an IC/BPS diagnosis compared to White respondents. Female gender and older age were significant predictors of diagnosis, while higher educational attainment was associated with increased odds of treatment receipt. Additional models adjusted for symptom severity (ICPI and ICSI indices) and current life circumstances, such as employment and insurance status, in predicting current treatment use. The Benjamini-Hochberg adjustment for multiple testing was applied, and adjusted *P* values are reported where applicable. These findings provide critical insights into potential disparities in IC/BPS diagnosis and treatment access, emphasizing the need for further investigation into barriers to care for underrepresented populations.

b The logistic regression predicting diagnosis with IC/BPS by a health care provider includes, besides ethnicity and race, other demographic characteristics such as gender assigned at birth, current age in years, as well as educational status. We hypothesized that having an immediate family member with an IC/BPS diagnosis and age at symptom onset influenced the likelihood of being diagnosed. Measures that reflect the current status of IC/BPS symptoms, such as the ICPI and the ICSI, were not included in this model as predictive variables because the diagnosis of IC/BPS occurred longer than 12 months ago. This applies also to variables describing the current employment status of the respondent as well as having had continuous health insurance in the past 12 months.

c The logistic regression models predicting if respondents ever received treatment in the past, if they self-treated in the past, and if they received prescription or in-office treatments in the past included the same demographic predictor variables as the logistic regression model predicting diagnosis: ethnicity, race, gender assigned at birth, current age in years, and education. In addition, we hypothesized that having a diagnosis by a health care provider would influence the receipt of treatment and therefore added this as a predictive variable to the model.

d The logistic model predicting if respondents receive current treatment should also be influenced by current symptom measures as well as current life circumstances. In addition to the demographic predictor variables from the previous models, such as ethnicity, race, gender assigned at birth, current age in years, and education, we added current employment status and having had continuous health insurance in the past 12 months. Besides having been diagnosed with IC/BPS by a health care provider, we added 2 current symptom measures, ICPI and ICSI, since worse symptoms should increase the likelihood of current treatment.

e OR: odds ratio.

f GED: general equivalency diploma.

g Not available.

h This *P* value is no longer statistically significant due to the new corrected significance level (*P*<.02) after the Benjamini-Hochberg adjustment for multiple testing.

i ICPI: Interstitial Cystitis Problem Index.

j ICSI: Interstitial Cystitis Symptom Index.

Having received a formal medical diagnosis of IC/BPS emerged as the strongest predictor of whether a participant had ever received any form of treatment for IC/BPS symptoms (OR 29.67, 95% CI 18.32‐48.05). Race and ethnicity were not independent predictors of treatment status in these models. However, those assigned female gender at birth had significantly higher odds than male gender of having ever received treatment (OR 2.24, 95% CI 1.31‐3.83) as well as having used self-treatment methods (OR 1.88, 95% CI 1.14‐3.10).

Self-reporting as a homemaker or student was associated with a significantly lower likelihood of currently receiving treatment compared to respondents employed full-time (OR 0.43, 95% CI 0.27‐0.69). Higher levels of educational attainment correlated with a greater likelihood of receiving treatment: compared to those with a high school education or less, those with a master's degree or higher had 2.07 times higher odds of ever using self-treatment methods (95% CI 1.13‐3.78) and 3.34 times higher odds of having received any form of treatment (95% CI 1.51‐7.39).

## Discussion

### Principal Findings

This large survey included individuals with diagnosed or suspected IC/BPS and documented their experiences across diagnostic and therapeutic landscapes. A central finding was that individuals who had IC/BPS symptoms and who identified as a racial or ethnic minority were substantially less likely to report having received a formal medical IC/BPS diagnosis from a health care provider than non-Hispanic White respondents, even after accounting for multiple potential confounding factors such as age, gender, age at symptom onset, educational attainment, and family history. This disparity in the rates of receiving a diagnosis appeared to drive other inequities observed in access to treatments requiring physician prescription or administration. Specifically, once the diagnostic status was controlled for in the multivariable analyses, racial and ethnic minorities were no longer statistically significantly less likely to receive any form of treatment for IC/BPS symptoms.

The survey data suggest that a major barrier to equal receipt of care is not inherently tied to race or ethnicity per se but rather stems from the reduced rates of diagnosis among minority patient populations. The definitive reasons underlying these diagnostic disparities could not be directly ascertained from this study but could possibly be due to individuals with suspected diagnoses reporting somewhat less severe symptomology as we observed, which may result in a lack of referral to a specialist or clinician who is familiar with the diagnosis of IC/BPS or other difficulties navigating the US health care system. Diagnostic disparities may also be due to a multitude of other factors, such as unconscious biases influencing medical decision-making [22-24], racism [25], poor communication [26], or differing abilities in advocating for health care needs [27]. Provider disbelief and pain dismissal have been reported in the female population diagnosed with IC/BPS [28], and people from racial and ethnic minority groups are less likely to have their pain taken seriously or treated [29,30], suggesting that this may also play a role. Regardless of the cause, this diagnostic gap represents the importance of intervention to achieve more equitable access to care across all patient demographic groups.

Compounding the issue of reduced diagnosis rates among minority or multiple-race individuals, this study also revealed gaps in the use of behavioral and nonpharmacologic treatments for IC/BPS. Compared to White and non-Hispanic individuals, minority or multiple-race and Hispanic participants reported lower rates of using several forms of self-treatment that could be implemented in an accessible, low-risk manner, such as dietary modifications, over-the-counter analgesics, anti-inflammatory agents, antihistamines, and nutritional supplements. Similarly, minority or multiple-race participants reported lower use rates of some prescription medications, such as antidepressants and pentosan polysulfate, as well as reduced exposure to commonly used interventional therapies, such as bladder instillations, hydrodistention, and pelvic floor physical therapy. Differences in the use of interventional therapies may be due to insurance coverage for these interventions; insurance type varied significantly between minority or multiple-race and White survey respondents and Hispanic and non-Hispanic respondents.

However, these discrepancies across a range of therapeutic options, many of which do not necessarily require formal IC/BPS diagnosis, highlight the need to improve awareness of and access to evidence-based treatment guidelines [1,8]. A recent study reported that the female population with IC/BPS has a strong interest in guided programs that teach self-care practices [31]. Campaigns could focus on easy-to-adopt therapies that have the potential to mitigate symptom burden prior to pursuing more invasive approaches [8].

The finding that over 25% (n=385) of the individuals who responded to the survey are using narcotic medications was striking and indicative of the persistent pain of this condition. Many patients remain symptomatic or undertreated (as evidenced by the ICSI/ICPI scores), despite multimodal therapy. Additionally, though there were meaningful differences in treatment use, there was no difference in ICSI/ICPI scores by race or ethnicity, possibly reflecting that treatments are not that effective overall.

### Limitations

Limitations of this study include the potential for biases inherent to the recruitment of participants via web-based surveys—such as recall and self-selection bias, which could limit the generalizability of the findings to the broader IC/BPS patient population. There is also the possibility that participants may have subjectively interpreted and responded to questions in a manner that introduced inconsistencies compared to objective clinical assessments. The survey did not ask any questions that can be a proxy for poor access to care or providers who may diagnose interstitial cystitis, such as having a primary care provider or getting routine screening. Additionally, despite targeted efforts to oversample racial and ethnic minority or multiple-race groups, the overall survey population was still underrepresented in these patient populations compared to the US demographic composition, which may have affected the power to detect differences between groups. Due to the small sample size, the ethnic minority or multiple-race groups were categorized together for analyses, which may have missed differences that would have been revealed by a more nuanced approach. The minority or multiple-race individuals were also younger than the White respondents, which may have impacted the results. While the age difference is consistent with the finding that Black and Hispanic individuals are more likely to receive a diagnosis of IC/BPS at a younger age [20], it may also be due to older minority or multiple-race individuals being less likely to use digital health information or access the internet [32].

Finally, because the survey was administered to all individuals self-identifying as having IC/BPS in a web-based community regardless of diagnosis status, it is possible that there was some degree of heterogeneity or imprecision in the extent to which undiagnosed respondents fully met the established clinical criteria for IC/BPS. However, IC/BPS is commonly misdiagnosed [7,20], indicating that restricting the sample to diagnosed individuals would not eliminate heterogeneity and imprecision. In addition, the “suspected diagnosis” respondents were more likely to be minority or multiple-race, so eliminating “suspected diagnosis” from the analyses would greatly reduce the diversity of individuals included in the study. It is important to note that the surveyed individuals with “suspected diagnoses” were still symptomatic, despite having reported milder symptoms. For example, the mean ICSI score of 10.3 (SD 3.52) for those with “suspected diagnosis” means that these individuals reported strong need to urinate about half the time, needing to urinate more than once per 2 hours, getting up 2 or more times a night, or experiencing pain or burning in their bladder fairly often.

### Conclusions and Future Directions

In this study of over 1600 respondents across diverse racial and ethnic backgrounds, a key disparity identified was the reduced likelihood of individuals who identified as racial or ethnic minority or multiple-race to have received a formal medical diagnosis of their condition compared to White individuals, even after controlling for multiple potentially confounding sociodemographic and clinical factors. This diagnostic gap appears to be a driver of downstream disparities in access to guideline treatments and therapies requiring clinical supervision or prescription. The findings indicate a need to focus on quality improvement efforts to facilitate accurate and timely IC/BPS diagnosis across all patient populations, regardless of race, ethnicity, or demographic characteristics. Initiatives to raise awareness of guideline-recommended and self-treatment approaches may reduce the identified disparities in the use of appropriate evidence-based therapies. Additional investigations into understanding the interplay between demographic factors and diagnosis are warranted.

## Abbreviations

IC/BPS: interstitial cystitis/bladder pain syndrome
ICA: Interstitial Cystitis Association
ICPI: Interstitial Cystitis Problem Index
ICSI: Interstitial Cystitis Symptom Index
OR: odds ratio
